# Studies of the role of steroid hormone in the regulation of oocyte maturation in cattle

**DOI:** 10.1186/1477-7827-4-4

**Published:** 2006-02-03

**Authors:** Hai Feng Wang, Naoki Isobe, Kanako Kumamoto, Hideaki Yamashiro, Yasuhisa Yamashita, Takato Terada

**Affiliations:** 1 Laboratory of Animal Reproduction, Graduate School of Biosphere Sciences, Hiroshima University, Higashi-Hiroshima, 739-8528, Japan

## Abstract

**Background:**

The objective of this study was to investigate whether the steroid hormone(s) secreted from cumulus-oocyte complexes (COCs) is a prerequisite for bovine oocyte maturation and cumulus expansion using aminoglutethimide (AGT), a P450 cholesterol side-chain cleavage inhibitor.

**Methods:**

In experiment 1, COCs were cultured in maturation medium with various concentrations of AGT for 22 h to determine the effective concentration of AGT to inhibit steroid hormone secretion, meiotic maturation and cumulus expansion. In experiment 2, COCs were cultured in conditioned medium (CM) and TCM-199 medium with or without 10 mM AGT to check whether steroid hormones secreted from COCs were responsible for oocyte maturation and cumulus expansion. Experiments 3 and 4 were carried out to determine whether exogenous progesterone or estradiol-17beta was able to overcome the inhibitory effects of AGT on oocytes maturation and cumulus expansion. COCs cultured in 10 mM AGT-containing medium supplemented with various concentrations of progesterone or estradiol-17beta for 22 h were examined for oocyte maturation and cumulus expansion.

**Results:**

Experiment 1 showed that a concentration of 10 mM AGT in medium was sufficient to block steroid hormone secretion, oocyte maturation and cumulus expansion, and that these inhibitory effects were fully reversible. In experiment 2, the addition of 10 mM AGT to CM did not significantly prevent oocyte maturation and cumulus expansion, implying that CM contains the steroid hormone(s) secreted from COCs, which are closely associated with oocyte maturation and cumulus expansion. The results in experiments 3 and 4 demonstrated that the addition of any concentration of progesterone or estradiol-17beta in the medium did not reduce the inhibitory effects of AGT on oocyte maturation and cumulus expansion.

**Conclusion:**

Our results indicate that bovine oocytes surrounded by cumulus cells are prevented from maturation and cumulus expansion through the inhibition of steroid secretion due to AGT, and that these inhibitory effects of AGT on oocyte maturation and cumulus expansions can not be overcome by the addition of either progesterone or estradiol-17beta in the medium. These observations suggest that some steroid hormone(s) other than P_4 _and E_2 _secreted from bovine COCs is essential for their meiotic maturation and cumulus expansion.

## Background

Mammalian oocytes are arrested at the dictyate stage until they have completed their growth phase. The resumption of meiosis is induced by the preovulatory gonadotropin surge in vivo or by transferring the oocytes from their surrounding somatic follicle cells into suitable culture conditions [1]. The nuclear and cytoplasmic events occurring during reinitiation of meiosis are called maturation and are the prerequisites for monospermic fertilization and early embryonic development. The maturation was controlled by many factors including steroid hormone in vivo and in vitro [[2,3] and [4]].

In vitro maturation of mammalian oocytes has been commonly performed using medium covered by paraffin oil or mineral oil. In previous studies using medium covered with oil, it has been reported that the addition of progesterone (P_4_) to the maturation medium stimulates meiotic re-initiation of bovine oocytes, regardless of the presence or absence of gonadal hormones [5], and that the addition of estradiol-17β (E_2_) with FSH in the maturation medium increases the maturation, morula, and blastocyst rates in bovine oocytes [6]. Furthermore, a detrimental effect of E_2 _on nuclear maturation [7] and cytoplasm maturation has been reported in porcine oocytes [8]. However, it has recently been demonstrated that the concentrations of E_2 _or P_4 _in medium are reduced in response to the high absorbing capacity of paraffin oil or mineral oil [9]. Therefore, it is thought that the effects of steroids on the maturation of oocytes cultured in medium covered by oil are unreliable.

On the other hand, addition of various concentrations of P_4 _to maturation medium without an oil covering has been found to have negative effects (P < 0.05) on the percentage of oocytes completing nuclear or cytoplasmic maturation in pig [10] and bovine oocytes [11]. Additionally, an established role of estradiol is to promote the change in calcium activity during cytoplasmic oocyte maturation [12], which in turn allows the typical calcium oscillation occurring during fertilization [13]. In contrast, it has been noted that E_2 _supplementation to a serum-free maturation medium negatively affects bovine oocyte meiotic resumption and subsequent embryo development [14].

It has recently been demonstrated that when COCs are cultured in medium supplemented with FSH and LH, they secrete P_4 _into the culture medium and the steroid enhances meiotic resumption of porcine oocytes [15]. Mingoti et al. [16] have also reported that cumulus cells of bovine COCs are able to secrete E_2 _and P_4 _in defined culture medium during in vitro maturation. The evidence reported in the literature suggests that the steroid hormones secreted from COCs during culture should be taken into consideration in assessing the effects of steroid hormones on oocyte maturation. However, this consideration has not been made in previous studies of the effects of adding steroid hormone to medium on oocyte maturation. Thus, the influence of exogenous P_4 _and E_2 _on in vitro maturation of mammalian oocytes should be reviewed. Since aminoglutethimide was known to inhibit the endogenous steroid production by the COCs [[17,18], and [19]], the effect of steroid hormone on the oocyte maturation should be evaluated using a AGT-containing medium.

The purposes of this study were as follows: (1) to determine the effective concentration of AGT to inhibit meiotic maturation, cumulus expansion, and steroid hormone secretion from bovine COCs; (2) to determine whether medium conditioned by COCs contains steroid hormone(s) secreted from COCs that are responsible for oocyte maturation and cumulus expansion; and (3) to test whether exogenous P_4 _or E_2 _can overcome the inhibitory effects of AGT on oocyte maturation and cumulus expansion.

## Materials and methods

### Oocyte recovery and culture conditions

Bovine ovaries were collected from a local slaughterhouse, and transported to the laboratory at 30°C in saline with 0.1 mg/ml kanamycin (Meiji Seika, Tokyo, Japan) in a thermos. The oocytes were then recovered by aspiration of antral follicles 2–8 mm in diameter using a 10-ml syringe with 21 5/8-gauage needles. Oocytes with compact unexpanded cumulus cells and with homogenous ooplasm were selected under a stereomicroscope and were placed in a watch glass containing the pre-warmed Dulbecco's phosphate-buffered saline (D-PBS) (pH 7.4) supplemented with 0.1% (w/v) Polyvinyl-pyrroli-done (PVP) (Sigma, St. Louis, MO, USA) and 100 μg/ml kanamycin. The basic culture medium was TCM-199 (Earle's salts Gibco. NY, USA) supplemented with 10% (v/v) heat-inactivated fetal calf serum (Gibco BRL, Grand Island, NY), 0.6 μg/ml porcine FSH (Sigma), 1.3 μg/ml equine LH (Sigma), and 50 μg/ml gentamicin. In order to avoid possible diffusion of the steroid into the oil, which would alter the effective concentration, cumulus-oocyte complexes (COCs) were transferred to 100 μl maturation medium in a 96-well multi-dish (Falcon, Franklin Lakes, NJ). All cultures were carried out at 39°C in a humidified atmosphere of 5% CO_2 _in air.

### Evaluation of nuclear maturation

After 22 h maturation, COCs were denuded by vortexing for 1 min in PBS supplemented with 0.1% (w/v) hyaluronidase (H3506; Sigma) and mounted on slides. The oocytes were fixed with acetic acid/ethanol (1:3) for 22 h, stained with 1% lacmoid in 45% acetic acid, and were examined under a phase-contrast microscope (400×) for evaluation of their chromatin configuration. An oocyte with either a polar body or 2 chromatin spots was defined as metaphase II.

### Assessment of cumulus expansion

The degree of cumulus expansion was assessed under a stereomicroscope after a 22-h incubation according to a subjective scoring system from 0 to +3 as follows: 0, no expansion; +1, separation of only the outermost layer of cumulus cells; +2, further expansion involving the outer half of the cumulus oophorus; +3, complete expansion including the corona radiate cells [20,21].

### Aminoglutethimide

Aminoglutethimide, an inhibitor of P450 cholesterol side-chain cleavage (AGT, Sigma), which could prevent the production of steroid hormones pregnenolone, 17-OH-pregnenolone, progesterone, 17-OH-progesterone, dehydroepiandrosterone (DHEA), androstenedione (delta4-dione), androst-5-ene-3 beta, 17beta-diol (delta5-diol), Testosterone, dihydrotestosterone (DHT), androstane-3 alpha, 17 beta-diol, androstane-3beta. 17beta-diol and 17beta-estradiol [24,25], was dissolved in dimethylsulfoxide (DMSO) (Sigma) at 1 mol/l and was stored at -20°C. Each final concentration was obtained by dilution in the maturation medium. As a control, inhibitor-free medium was prepared by adding 0.098% (v/v) DMSO to the basic maturation medium. This concentration of DMSO has no adverse effects on oocyte maturation in bovines [26].

### Preparation of COCs-conditioned medium (CM)

TCM-199 medium supplemented with 10% (v/v) FCS, 0.6 μg/ml FSH, and 1.3 μg/ml LH was conditioned by 5 COCs with 100μl maturation medium in 96-well plates at 39°C in a humidified atmosphere of 5% CO_2 _in air. The conditioned medium was then aspirated from the well and subjected to centrifugation at 10000 × *g *for 20 min to remove cells and other debris, and was either used immediately for IVM or frozen at -20°C until use.

### Preparation of medium with Progesterone and Estradiol-17β

Progesterone (P_4_) and estradiol-17β(E_2_) (Sigma) stock solution was dissolved in ethanol (Katayamakagaku, Osaka, Japan) at a concentration of 1 mg/ml. Further dilutions (100 fold) were made with ethanol to achieve a concentration of 10 μg/ml. The ethanol was evaporated, and maturation medium was added to achieve the stock concentration of 2 μg/ml and stored at -20°C. Each final concentration was obtained by dilution with the maturation medium.

### Measurements of Progesterone, Estradiol-17β, and Testosterone in medium

All corresponding supernatants of COCs were measured for concentrations of P_4_, E_2_, and testosterone. An enzyme immunoassay (EIA) on 96-well ELISA plates (SUMILON, NY, USA) coated with the second antibody was performed as described previously [22,23]. The sensitivities, intra-assay, and inter-assay CV were 5.5 pg/ml, 8.6–8.7% and 12.2–12.7% for progesterone, 16 pg/ml, 8.5–9.3% and 9.5–10.5% for estradiol-17β and 7.0 pg/ml, 6.1–6.9% and 6.5–9.1% for testosterone, respectively.

### Experimental designs

#### Experiment 1

To determine which level of AGT is effective to inhibit the bovine oocyte maturation, cumulus expansion, and steroid hormone secretion, five COCs were cultured in 100 μl maturation medium supplemented with 0, 0.25, 0.5, 1.0, and 10 mM AGT for 22 h. After the degree of cumulus expansion was assessed under a stereomicroscope, the denuded oocytes were fixed to examine their nuclear stages. The medium was collected into plastic tubes and centrifuged at 10 000 × g for 20 min. The resulting supernatant was stored at -80°C until assay by EIA.

In the reversibility experiments, which confirmed the meiotic competence of oocytes treated with high concentrations of AGT, the collected COCs were divided into two equal parts. Half of the COCs were cultured for 22 h in maturation medium without AGT as a control. The remainder was cultured in the medium with 10 mM AGT, and then further cultured for 22 h in the medium without AGT. At the end of the cultivation period, these oocytes were assessed wither regard to their nuclear maturation and cumulus expansion as described above. This experiment was replicated more than four times on different days and a total of 120–140 COCs were assayed for each treatment. Five COCs as a group were cultured in 100 μl maturation medium per well on multi-dish. If one COC in the group was lost, the other oocytes of the group were not taken into account.

#### Experiment 2

This experiment was conducted with the aim of checking if the factors, (particularly steroid hormone) directly associated with oocyte maturation and cumulus expansion were secreted from COCs. COCs were cultured in conditioned medium with or without 10 mM AGT for 22 h, and the control oocytes were cultured in TCM-199 with or without 10 mM AGT. After cumulus expansion of COCs was assessed at the end of cultivation with a stereomicroscope, oocytes were denuded and fixed, and their nuclear status was evaluated. The experiment was replicated at least three times on different days and a total of 120–130 COCs were assayed for each treatment.

#### Experiment 3

This experiment was carried out to determine whether exogenous P_4 _was able to overcome the inhibitory effects of AGT on oocyte maturation and cumulus expansion. COCs were cultured in maturation medium containing with 10 mM AGT and various concentrations of P_4 _(0, 100, 500, and 1000 ng/ml) for 22 h. After IVM culture, cumulus expansion and nuclear status were evaluated. The experiment was replicated at least three times on different days and a total of 100–145 COCs were assayed for each treatment.

#### Experiment 4

The intent of this experiment was to investigate whether exogenous E_2 _can overcome the inhibitory effect of AGT on oocyte maturation and cumulus expansion. COCs were cultured in 10 mM AGT-containing medium supplemented with various concentrations of E_2 _(0, 100, 500, and 1000 ng/ml). After the cultivation of COCs for 22 h, cumulus expansion and the nuclear status were evaluated as in experiment 3. The experiment was replicated at least three times on different days and a total of 90–100 COCs were assayed for each treatment.

### Statistical analysis

Statistical analyses were carried out with one-way ANOVA followed by the Duncan multiple range test using StatView software 5.0 (Abacus Concepts Inc., Berkeley, CA, USA). All percentage data were subjected to an arcsine transformation before statistical analysis. Data from three replicates were expressed as mean ± SEM. A probability of P < 0.05 was considered to be significant.

## Result

### Experiment 1

**Figure 1:**
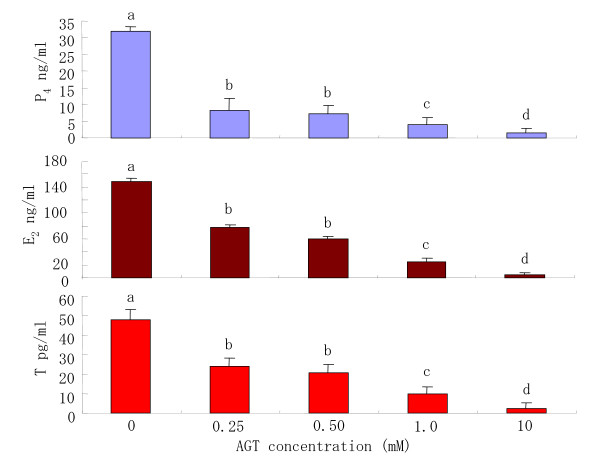
Steroid hormones secreted by COCs that were incubated in the medium with various concentrations of aminoglutethimide (AGT). ^a–d^Different superscripts show significant differences (P < 0.05). Data are mean ± SEM from three different experiments. P_4_: progesterone; E_2_: estradiol-17beta; T: testosterone.

In order to determine the sufficient concentration of aminoglutethimide (AGT) in maturation medium to inhibit steroid hormone, COCs were cultured in medium with various concentrations of AGT for 22 h. The concentrations of P_4_, E_2 _and testosterone in the maturation medium were significantly decreased with increased concentrations of AGT added to the maturation medium (Fig 1). When COCs were cultured in maturation medium with 10 mM AGT for 22 h, the P_4_, E_2 _and testosterone secretion were almost completely inhibited.

**Figure 2:**
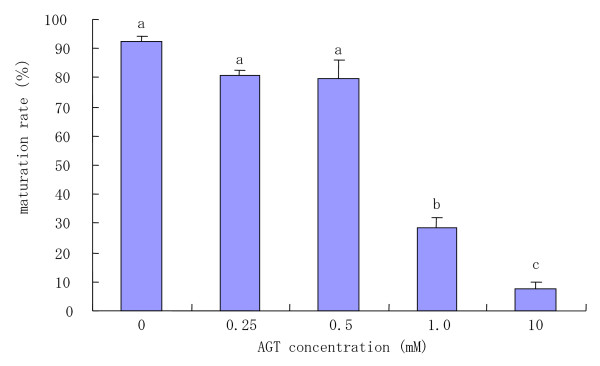
Maturation rates of COCs cultured in medium with various concentrations of AGT. ^a–c^Different superscripts show significant differences (P < 0.05). Data are mean ± SEM from three different experiments. AGT: aminoglutethimide.

When COCs were cultured in medium with various concentrations of AGT for 22 h to determine the effect of AGT on the oocyte maturation, there was a marked decrease in the proportion of matured oocytes as the concentrations of AGT increased (Fig 2). The inhibitory effects of AGT reached a plateau, and no significant difference was found between 0.25 and 0.5 mM AGT treatments. The addition of 1.0 mM AGT led to a significant (P < 0.05) decrease in the proportion of matured oocytes. Moreover, the addition of 10 mM AGT led to an almost complete block of oocyte nuclear maturation.

The percentage of COCs with +3 expansions decreased with increased concentrations of AGT in medium (Table 1). When 10 mM AGT was added to the maturation medium, only COCs exhibiting a 0 or +1 degree of cumulus expansion was observed.

**Table 1:** Cumulus expansion of COCs cultured in medium with various concentrations of AGT

		Cumulus expansion (%)			
		
Concentration of AGT (mM)	Number of Oocytes examined	0	+1	+2	+3
Control	120	0^a^	12.4 ± 1.3^c^	25.0 ± 4.9^e^	62.5 ± 4.0^g^
0.25	120	0^a^	10.9±1.4^c^	26.7±0.9^e^	62.4±1.7^g^
0.50	120	0^a^	15.8 ± 3.6^c^	34.0 ± 3.1^e^	50.2 ± 1.6^g^
1.0	130	0^a^	50.8 ± 4.2^d^	38.4 ± 3.3^e^	10.8 ± 2.1^h^
10.0	140	76.8 ± 2.2^b^	23.2 ± 2.2^c^	0^f^	0^i^

^a-b, c–d, e–f, g–i^ : Columns with no common superscripts were significantly different (P < 0.05). Values are mean ± SEM of three different replicates. Control: COCs were incubated in medium without addition of AGT; AGT: aminoglutethimide.

When COCs treated with 10 mM AGT for 22 h were cultured for an additional 22 h in maturation medium without 10 mM AGT, the proportion of matured oocytes and the percentage of COCs with +3 expansions were not significantly different from those of the control, suggesting that the inhibitory effects of AGT on meiotic maturation and cumulus expansions are fully reversible (Table 2).

**Table 2:** Maturation rate and cumulus expansion of COCs incubated further for 22-h in medium without AGT after 22-h cultivation in medium with 10 mM AGT

Treatment	Number of Oocytes examined	Maturation rate (%)	Cumulus Expansion +3 (%)
Control^1)^	120	92.3 ± 1.6	79.4 ± 4.0
AGT-10 mM^2)^	120	89.4 ± 4.5	78.7 ± 5.7

^1) ^Oocytes were cultured for 22 h in TCM-199. ^2) ^Oocytes were cultured in TCM-199 medium with 10 mM AGT for 22 h and then COCs were further cultured 22 h in TCM-199 medium without 10 mM AGT. Values are mean ± SEM of three different replicates. AGT: aminoglutethimide.

### Experiment 2

**Figure 3:**
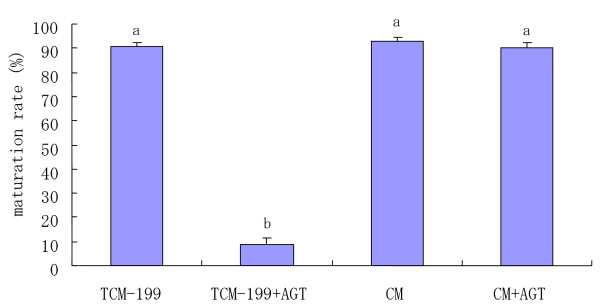
Maturation rates of COCs incubated in conditioned medium and TCM-199 medium with or without 10 mM AGT. ^a–b^Different superscripts show significant differences (P < 0.05). Data are mean ± SEM from three different experiments. CM: conditioned medium; AGT: aminoglutethimide.

To determine whether the factors (particularly steroid hormones) secreted from COCs associated with oocyte maturation existed in conditioned medium (CM), COCs were cultured in CM and TCM-199 with or without 10 mM AGT for 22 h. The proportion of matured oocytes cultured in CM with 10 mM AGT was significantly (P < 0.05) higher than that in TCM-199 with 10 mM AGT(Fig 3). However, there were no significant differences among the proportions of matured oocytes in the TCM-199 medium, CM and CM+AGT. When COCs were matured in CM with 10 mM AGT, a significantly higher percentage of COCs with +3 expansions was observed as compared with that of COCs inTCM-199 with 10 mM AGT (Table 3).

**Table 3:** Cumulus expansion of COCs incubated in conditioned medium and TCM-199 medium with or without 10 mM AGT

		Cumulus expansion (%)			
		
Treatment	Number of Oocytes examined	0	+1	+2	+3
TCM	120	0^a^	4.4 ± 3.5^c^	39.7 ± 3.5^e^	55.9 ± 1.7^g^
TCM+AGT	120	75.3 ± 1.8^b^	24.7 ± 1.8^d^	0^f^	0^h^
CM	120	0^a^	5.3 ± 4.3^c^	31.4 ± 1.9^e^	63.3 ± 3.9^g^
CM+AGT	130	0^a^	6.3 ± 2.8^c^	33.8 ± 5.5^e^	59.9 ± 1.3^g^

^a-b, c–d, e–f, g–h^ : Columns with no common superscripts were significantly different (P < 0.05). Values are mean ± SEM of three different replicates. TCM: TCM-199 medium; CM: conditioned medium; AGT: aminoglutethimide.

### Experiment 3

**Figure 4:**
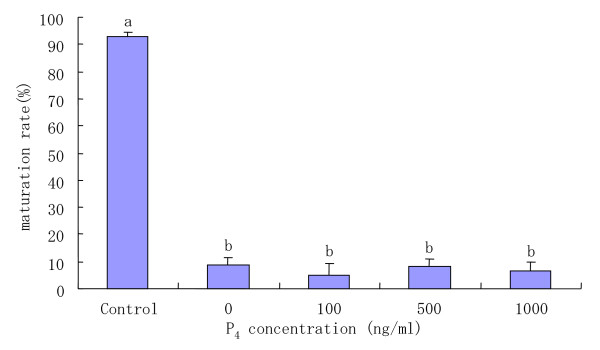
Maturation rates of COCs incubated with 10 mM AGT-containing medium supplemented with various concentrations of P_4_. ^a–b^Different superscripts show significant differences (P < 0.05). Data are mean ± SEM from three different experiments. Control: COCs were incubated in medium without addition of AGT. P_4_: progesterone; AGT: aminoglutethimide.

The purpose of this experiment was to examine whether or not progesterone in the medium would overcome the inhibitory effects of AGT on oocyte maturation. When COCs were cultured in medium with 10 mM AGT, the proportion of matured oocytes was significantly (P < 0.05) decreased compared with that of control oocytes cultured without AGT and P_4 _(Fig 4). When COCs were cultured in various concentrations of P_4 _and 10 mM AGT for 22 h, there were no significant differences in the proportion of matured oocytes among any concentration of P_4_. Similarly, no significant differences in the percentage of COCs exhibiting a +3 degree of cumulus expansion were found after 22 h of culture in medium with any concentrations of P_4 _and 10 mM AGT (Table 4).

**Table 4:** Cumulus expansion of COCs incubated in the 10 mM AGT-containing medium supplemented with various concentrations of P_4_

		Cumulus expansion (%)			
		
Concentration of P_4 _(ng/ml)	Number of Oocytes examined	0	+1	+2	+3
Control	100	0^a^	19.2 ± 6.0	20.1 ± 4.9^c^	60.7 ± 7.7^e^
0	110	84.9 ± 5.5^b^	15.1 ± 5.5	0^d^	0^f^
100	105	90.2 ± 3.5^b^	9.8 ± 3.5	0^d^	0^f^
500	115	87.5 ± 1.2^b^	12.5 ± 1.2	0^d^	0^f^
1000	145	89.5 ± 1.3^b^	10.5 ± 1.3	0^d^	0^f^

^a-b, c–d, e–f^Columns with no common superscripts were significantly different (P < 0.05). Values are mean ± SEM of three different replicates. Control: COCs were incubated in medium without addition of 10 mM AGT and P_4_; AGT: aminoglutethimide; P_4_: progesterone.

### Experiment 4

**Figure 5:**
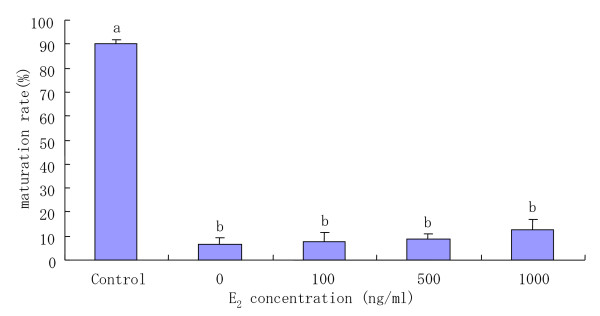
Maturation rate of COCs incubated with 10 mM AGT-containing medium supplemented with various concentrations of E_2_. ^a–b^Different superscripts show significant differences (P < 0.05). Data are mean ± SEM from three different experiments. Control: COCs were incubated in medium without addition of AGT. E_2_: estradiol-17beta; AGT: aminoglutethimide.

COCs were cultured for 22 h in the medium containing various concentrations of E_2 _and 10 mM AGT in order to explore the possibility that the addition of E_2 _in medium with 10 mM AGT could overcome the inhibitory effects of AGT on oocyte maturation. The proportion of matured oocytes after 22 h of culture in the presence of 10 mM AGT was significantly (P < 0.05) lower than that of control in the absence of 10 mM AGT (Fig 5), and there were no significant differences in maturation rates among the treatments with any concentrations of E_2_. Further, COCs cultured in 10 mM AGT-containing medium with various concentrations of E_2 _exhibited only a 0 or +1 degree of cumulus expansion (Table 5).

**Table 5:** Cumulus expansion of COCs incubated in the 10 mM AGT-containing medium supplemented with various concentration of E_2_

		Cumulus expansion (%)			
		
Concentration of E_2 _(ng/ml)	Number of Oocytes examined	0	+1	+2	+3
Control	100	0^a^	16.2 ± 4.7	25.2 ± 5.0^c^	58.6 ± 4.3^e^
0	95	89.8 ± 4.1^b^	10.2 ± 4.1	0^d^	0^f^
100	90	88.4 ± 3.9^b^	11.6 ± 3.9	0^d^	0^f^
500	100	85.9 ± 4.0^b^	14.1 ± 4.0	0^d^	0^f^
1000	95	91.7 ± 0.3^b^	8.3 ± 0.3	0^d^	0^f^

^a–b, c–d, e–f^Columns with no common superscripts were significantly different (P < 0.05). Values are mean ± SEM of three different replicates. Control: COCs were incubated in medium without addition of 10 mM AGT and E_2_; AGT: aminoglutethimide; E_2_: estradiol-17beta.

## Discussion

The results of the present study have demonstrated that the maturation and cumulus expansion of bovine oocytes surrounded by cumulus cells were prevented from maturation and cumulus expansion through the inhibition of steroid secretion due to 10 mM AGT, and that these inhibitory effect of AGT on oocytes maturation and cumulus expansion can not be overcome by the addition of either P_4 _or E_2 _in maturation medium.

Concerning of AGT concentration, Lieberman et al. [17] have reported that when rat oocytes are cultured in maturation medium with 1.0 mM AGT, the progesterone secretion is reduced to approximately 5% of that in controls without AGT. Moor et al. [27] have also reported that only 7% progesterone secretion in controls was observed in sheep COCs cultured in maturation medium with 1 mM AGT. Further, the progesterone secretion from porcine COCs cultured in maturation medium with 1 mM AGT was decrease to approximately 5% of control levels [15]. In the present study, the progesterone secretion form bovine COCs cultured in maturation medium with 1 mM AGT declined to approximately 4% of control levels (Fig 1). Thus, it is assumed that AGT sensitivity to progesterone secretion from COCs is highly comparable among these animal species.

In the present study, approximately 30% of bovine oocytes cultured in medium with 1.0 mM AGT did not complete meiotic maturation (Fig 2). Shimada and Terada [15] have also indicated that the proportion of matured porcine oocyte cultured in medium with 1.0 mM AGT was decreased to approximately 30%. In contrast, it was found that when rat COCs were cultured in medium with 1.0 mM AGT, more than 90% of the oocytes underwent GBVD [17]. Similar results were obtained by Moor [27], who showed that the addition of 1.0 mM AGT to medium does not affect meiotic maturation in sheep COCs. These findings clearly show that the sensitivity of oocyte maturation for AGT in culture medium is different among animal species. Therefore, it is conceivable that although the AGT sensitivity of COCs with regard to maturation is dependent on the animal species, the AGT sensitivity of cumulus cells surrounding oocytes for progesterone secretion is highly comparable among animal species, as described above. As such, the role that steroid hormones play in oocyte maturation may differ significantly according to the animal species.

When oocytes were cultured in medium with 10 mM AGT for 22 h, only 10% of oocytes reached the MII stage, and steroid hormone secretion (P_4 _or E_2_) at approximately 5% of control levels was detected in maturation medium, suggesting that steroid hormone(s) secreted from COCs was responsible for oocyte maturation. The cumulus expansion was also completely inhibited in the presence of 10 mM AGT. From these data, it was demonstrated that AGT concentrations of 10 mM AGT are sufficient to inhibit oocyte maturation, steroid secretion, and cumulus expansion in bovine oocytes.

Furthermore, when oocytes cultured in medium with 10 mM AGT for 22 h were further incubated for 22 h in medium without 10 mM AGT, the proportion of oocytes reaching the MII stage and the percentage of COCs with +3 degree expansions were equal to the untreated control group. These results indicate that bovine COCs incubated in medium with 10 mM AGT for 22 h have their full potential to undergo meiotic maturation and cumulus expansion.

Compared to the TCM-199 medium with 10 mM AGT, a significantly higher proportion of matured oocytes and the percentage of COCs with +3 degree expansions were observed in CM with 10 mM AGT (Fig 3). This result suggests that steroid hormone(s) secreted in medium from COCs can overcome the inhibitory effect of AGT on oocyte maturation and cumulus expansion. This finding is also supported by the observation that no significant differences in the proportion of matured oocytes and the percentage of COCs with +3 degree expansions were seen among CM with AGT, CM without AGT, and TCM-199 medium without AGT.

It is well-established that E_2 _and P_4 _in mammalian preovulatory follicles are the primary steroids secreted from granulosa cells stimulated by FSH or LH [19,28]. Several studies have also shown that steroids may be involved in the control of oocyte maturation [[5,15,29] , and [30]]. Mingoti et al. [16] have provided evidence that cumulus cells surrounding bovine COCs secrete estradiol and progesterone in culture systems for in vitro maturation. Similarly, the results of the present study indicate that maturation medium without AGT in which COCs were cultured for 22 h contained significantly high concentrations of P_4 _and E_2_, but very low concentrations of testosterone (Fig 1). On the basis of the combined evidence from the literature and our results, we attempted to determine whether exogenous P_4 _or E_2 _is a key prerequisite for oocyte maturation. However, the results demonstrated that the addition of any concentrations of P_4 _or E_2 _in the culture medium did not overcome the inhibitory effect of AGT on meiotic maturation (Figs 4, 5). These results led us to conclude that P_4 _and E_2 _are not the essential steroid hormones secreted from COCs to promote bovine oocyte maturation and cumulus expansion.

Many of the factors secreted from COCs stimulated by gonadotrophin have been reported to affect the meiotic maturation of oocytes [31,32]. One of the maturation factors, Meiosis-activating sterols (MAS), which was first purified from human follicular fluid and identified as 4, 4-dimethyl-5α-cholest-8, 14, 24-trien-3β-ol, has been shown to be synthesized by COCs in response to FSH stimulation [3]. It has also been suggested in cattle that FF-MAS addition in maturation medium can induce in vitro meiotic maturation of oocytes [33,34]. Meanwhile, it is likely that testosterone acts as the primary physiologic regulator of oocyte maturation in X. laevis [35,36]. Testosterone-stimulated resumption of meiosis in mouse oocytes has also been observed by Gill et al. [37], and these authors have claimed that dominant ovarian follicles in mammals may produce sufficient levels of androgens to override the constitutive inhibitory signals for maturation. These findings suggest that the MAS and testosterone secreted from cumulus cells surrounding bovine oocytes into the medium may be involved in their maturation and cumulus expansion. Since AGT inhibit the endogenous secretion of testosterone from COCs in the present study (Fig 1), it is highly probable that testosterone is a prerequisite for bovine oocyte maturation.

Thus, the physiological effects of both androgen and MAS on bovine oocyte maturation and cumulus expansion are now under investigation using culture medium with AGT.

In summary, the results of the present study have demonstrated that bovine oocytes surrounded by cumulus cells are prevented from maturation and cumulus expansion through the inhibition of steroid secretion due to AGT, and that these inhibitory effects of AGT on oocytes maturation and cumulus expansion can not be overcome by the addition of either P_4 _or E_2 _in maturation medium. It is therefore concluded that some steroid hormone(s) other than P_4 _and E_2 _secreted from bovine COCs is essential to their meiotic maturation and cumulus expansion.

## Authors' contributions

HFW was responsible for the design, coordination of the study, and experiments He performed oocyte cultivation and hormonal measurements, and participated in the analysis of data and in drafting the manuscript. NI collaborated in the hormonal measurements. KK, HY and YY collaborated in oocyte maturations. TT was responsible for the design and coordination of the study. He analyzed the data and drafted the manuscript. All authors read and approved the final manuscript.
